# Viability of Microencapsulated Probiotics in Cross-Linked Alginate Matrices and Chia Seed or Flaxseed Mucilage During Spray-Drying and Storage

**DOI:** 10.3390/microorganisms13071457

**Published:** 2025-06-23

**Authors:** Mariela Bustamante, B. Dave Oomah, César Burgos-Díaz, Daniela Vergara, Liset Flores, Carolina Shene

**Affiliations:** 1 Department of Chemical Engineering, BIOREN, Center of Food Biotechnology and Bioseparations, Universidad de La Frontera, Temuco 4811230, Chile; liset.flores@ufrontera.cl (L.F.); carolina.shene@ufrontera.cl (C.S.); 2 Centre of Biotechnology and Bioengineering (CeBiB), Universidad de La Frontera, Temuco 4811230, Chile; 3 Centro de Excelencia en Investigación Biotecnológica Aplicada al Medio Ambiente (CIBAMA), Universidad de La Frontera, Temuco 4811230, Chile; 4 Summerland Research and Development Centre, Agriculture and Agri-Food Canada, Summerland, BC V0H 1Z0, Canada; oomahd@gmail.com; 5 Agriaquaculture Nutritional Genomic Center (CGNA), Temuco 4780000, Chile; cesar.burgos@cgna.cl; 6 Laboratory of Pharmaceutical and Cosmetic Bioproducts, Center of Excellence in Translational Medicine—Scientific Technological Bioresource Nucleus (CEMT-BIOREN), Faculty of Medicine, Universidad de La Frontera, Temuco 4811230, Chile; daniela.vergara@ufrontera.cl; 7 Millennium Nucleus Bioproducts, Genomics and Environmental Microbiology (BioGEM), Avenida España 1680, Valparaíso 2390123, Chile

**Keywords:** probiotics, mucilage, chia seed, flaxseed, cross-linked alginate matrices

## Abstract

Interest in probiotics has not diminished, and techniques to protect them from the environment in which they are found are constantly being innovated. Spray-drying is the most studied and industrially used technique to encapsulate probiotics. Recently, a new process has been developed in which particle formation, alginate cross-linking, and drying are carried out in a single step. In this study, *Bifidobacterium infantis*, *Bifidobacterium longum*, *Lactobacillus plantarum*, and *Lactobacillus rhamnosus* were microencapsulated by spray-drying using a cross-linked alginate matrix supplemented with chia seed mucilage (CM) or flaxseed mucilage (FM) as the coating material. All formulations evaluated, supplemented with 0.4% (*w/v*) of CM or FM, including the control formulation showed high survival rates, varying between 87% and 97%. The viability of microencapsulated probiotics was affected by storage temperature. At 4 °C, viability decreased slightly, and after 90 days, the viable probiotic count ranged from 7 to 11 Log CFU/g of dry powder. Meanwhile, viability did not exceed 4 Log CFU/g of dry powder at 37 °C. Probiotic microencapsulation in cross-linked alginate matrices and chia or flaxseed mucilage by spray-drying is presented as a promising alternative for their protection, potentially improving the long-term stability and efficacy of the probiotic product.

## 1. Introduction

The interest in lactic acid-producing bacteria has not waned since Élie Metchnikoff (1908 Nobel Prize in Physiology or Medicine) observed that the consumption of these microorganisms beneficially affected host/human health. The probiotics market is expected to reach USD 84.85 billion in 2025 and USD 121.99 billion by 2030, with a compound annual growth rate (CAGR) of 7.53% during the forecast period (2025–2030) [1]. Several studies demonstrate that probiotic consumption has health benefits for the host and that each probiotic strain has specific properties that can alleviate diabetes mellitus and irritable bowel syndrome, reducing the risk or duration of upper respiratory tract infections, among others. Overall, the probiotic market is segmented into probiotic foods, probiotic drinks, dietary supplements, and animal feeds. Of these, the one with the greatest consumer acceptance is the dietary supplements, such as capsules, tablets, gummies, chewables, and powdered supplements [1].

Probiotics have been defined as “Live microorganisms which, when administered in adequate amounts, confer a health benefit on the host” [2]. Therefore, probiotics are non-pathogenic microorganisms, where *Bifidobacterium* and *Lactobacillus* genera are the most studied and used in the food, pharmaceutical, and cosmetic areas. The beneficial effects of bifidobacterial consumption on human health have been mainly associated with the prevention and treatment of gastrointestinal disorders (intestinal infections and cancer) [3]. Furthermore, they have neuroprotective effects that may delay the progression of Alzheimer’s disease and Parkinson’s disease [4], help in the prevention and treatment of depression [5], and improve motor symptoms and related digestive complications in patients with Parkinson’s disease [6]. The presence of *B. infantis* in the gastrointestinal tract of infants has been linked to accelerated maturation of the immune system, modulation of immune responses to suppress inflammation, and improved gastrointestinal barrier function [7]. On the other hand, *B. longum* 1714 consumption improves sleep quality and social functioning and increases energy/vitality because this strain modulates neural activity that correlates with improved vitality/reduced mental fatigue [8]. *Lactiplantibacillus plantarum*, known previously as *Lactobacillus plantarum* [9], promotes balance and improves host intestinal microbiota [10,11], improving mood, synaptic ability, depression, and cognitive ability. It has also been used to treat chronic and cardiovascular diseases such as Alzheimer’s, Parkinson’s, and others [12]. On the other hand, *Lacticaseibacillus (*synonym: *Lactobacillus) rhamnosus* exerts beneficial effects on the composition of the human gastrointestinal microbial community and the immune system [13].

Cell viability of both genera, *Bifidobacterium* and *Lactobacillus*, is affected by pH, processing and storage temperatures, salt and oxygen concentration. Foods or supplements should contain at least 6–7 Log CFU/mL or Log CFU/g at the time of consumption [14], a concentration that enables probiotics to survive gastrointestinal transit and reach the small intestine in sufficient quantities to exert the expected effects. In this context, microencapsulation can protect probiotics from adverse conditions, reducing loss of viability and maintaining their metabolic activity. Spray-drying and freeze-drying are the most used drying techniques for microencapsulation. Freeze-drying is a conventional method characterized by low production yields and long drying times. In contrast, spray-drying is easily scalable, offers high production rates, and can be up to ten times more cost-effective than freeze-drying [15]. Although spray-drying has several advantages, high drying temperatures can affect the viability of probiotics due to damage to the cell membrane. One option to reduce thermal damage is through the choice of an appropriate coating or wall material. Several coating materials are available for probiotics microencapsulation, such as inulin, maltodextrin, whey protein, flaxseed mucilage (FM), chia seed mucilage (CM), chitosan, prebiotics, and others. CM, FM, and inulin were used as a coating material to evaluate the possibility of increasing *Lactobacillus casei* var. *rhamnosus* survival during spray-drying. The conditions that maximized *L. rhamnosus* survival (90%) predicted by a face-centered central composite design were maltodextrin (14.4%, *w/v*) supplemented with CM (0.6%, *w/v*) and an inlet air temperature of 90 °C [16].

The demand for natural, safe, sustainable, and biodegradable products that not only provide nutritional value but also offer technological functionalities—such as structuring, texturizing, and stabilizing—is steadily increasing. In this context, mucilages have emerged as a promising alternative due to their properties as thickening, emulsifying, and gelling agents. Additionally, they are associated with various health benefits, including the modulation of postprandial glycemia and the regulation of the intestinal microbiota, among others [17,18,19].

Today, Chia (*Salvia hispanica* L.) seed is of interest due to its nutritional properties and beneficial effect on human health. Chia seeds exude a gel or mucilage in the presence of water, which represents about 6% of chia seeds, composed mainly of monosaccharides (85%) [20]. It also contains planteose, a galactosyl-sucrose oligosaccharide, a prebiotics component that stimulates the growth of beneficial bacteria and prevents the growth of pathogenic bacteria in the gastrointestinal tract [21]. CM can reduce the glycemic index of foods and regulate satiety [17,22,23]. Furthermore, it exhibits functional characteristics such as water-absorption capacity, emulsifying and foaming properties [18], and is photostable after 2 h under UV light [24].

Flaxseed or linseed (*Linum usitatissimun* L.) is a functional food ingredient due to its human health benefits. Like chia seed, flaxseed exudes a gel when in contact with water, which can represent about 6% of the seed [25]. FM is a heterogeneous polysaccharide containing two types of polysaccharides: an acidic pectic-like material and neutral arabinoxylan [26]. Acid polysaccharides present rhamnose, an indigestible oligosaccharide, considered a prebiotic that can improve probiotic viability [19]. The soluble fiber from FM delays gastric emptying improves glycemic control, and protects the mucosa of the gastrointestinal tract. It also lowers fasting blood sugar levels and total cholesterol levels, especially low-density lipoprotein cholesterol in type 2 diabetics [19]. FM is of interest to the food, pharmaceutical, and cosmetic industries because it can be used as a thickening, emulsifying, and mucoadhesive agent and drug release retardant [27].

Traditionally, probiotic microencapsulation with sodium alginate is prepared by extruding or spraying a sodium alginate solution with probiotics into a calcium chloride solution under constant and homogeneous agitation to finally preserve the capsules in a moist medium or dry. This method is difficult to scale up, and the particle size (≥300 μm) limits its application [28]. In contrast, spray-drying is a continuous, single-step, scalable, reproducible process that generates a low moisture product and 1–60 μm particle size that facilitates its incorporation into food, cosmetics, and pharmaceutical products [29,30]. A new method has been proposed to produce cross-linked alginate microcapsules by spray-drying. This method involves feeding a sodium alginate solution containing an insoluble calcium salt, an organic acid, and a volatile base into the spray dryer. After the fluid is atomized into droplets by the nozzle, the base evaporates because of the hot air stream, resulting in acidification of the droplets, which solubilizes the calcium salt and cross-links the alginate in one step [31]. Several studies have shown that probiotics microencapsulation by spray-drying produces powders with high viability after processing. However, no studies to date have evaluated the probiotic viability after spray-drying microencapsulation in cross-linked alginate matrices supplemented with CM or FM.

The aim of this study was to determine the effect of a coating material composed of a cross-linked alginate matrix supplemented with CM or FM on the viability of *B. infantis*, *B. longum*, *L. plantarum*, and *L. rhamnosus* after spray-drying and during storage. In addition, the stability of the powders during storage was evaluated.

## 2. Materials and Methods

### 2.1. Media and Cultivation Conditions

*Bifidobacterium infantis* ATCC15679, *Bifidobacterium longum* ATCC15707, *Lactobacillus rhamnosus* ATCC53103, and *Lactobacillus plantarum* ATCC8014 (American Type Culture Collection, Rockville, MD, USA) were grown on MRS broth (BD, Baltimore, MD, USA) [32]. Each probiotic strain was sub-cultured thrice with 5% (*v*/*v*) inoculum in 5 mL of MRS broth and incubated (37 °C, 12 h). For strains of the *Bifidobacterium* genus, MRS broth was combined with L-cysteine-HCl (0.05%, *w/v*) and incubated under anaerobic conditions with GasPaK^TM^ EZ (anaerobiosis generator system) (BD, Baltimore, MD, USA).

For the encapsulation assays, MRS (200 mL) was inoculated with 5% (*v*/*v*) of the grown pre-culture and incubated (37 °C, 12 h). Then, the probiotic biomass was recuperated by centrifugation (6000× *g*, 4 °C, 15 min); the probiotic biomass was washed twice with sterile distilled water and centrifuged as described above. Finally, probiotic biomass was re-suspended in sterile distilled water (2 mL) and added to the encapsulating solution.

### 2.2. Extraction of the Chia Seed and Flaxseed Mucilage

Chia seed and flaxseed were purchased from the local market. CM was extracted according to Bustamante et al. [16]. Briefly, seeds were extracted with hot distilled water (80 °C, pH 5.0, 2 h) at a 1:40 (*w*/*v*) ratio. The extraction cycle was repeated twice.

FM was extracted according to Bustamante et al. [33]. Seeds were extracted with hot distilled water (90–95 °C, pH 5.0, 30 min) at a 1:10 (*w*/*v*) ratio. The extraction cycle was repeated three times.

Both CM and FM extracts were separated from the seeds and spread on trays to be dried in an air convection oven (60 °C), milled, sieved (0.425 mm mesh), and stored (−20 °C) until use.

### 2.3. Control Encapsulating Solution Preparation

The control encapsulation solution was formulated in 120 mL distilled water: 100 mL were prepared with low viscosity sodium alginate (4.0%, *w/v*) (Sigma-Aldrich, St. Louis, MO, USA) and succinic acid (2.0%, *w/v*) (Merck, Darmstadt, Germany). After sterilization (121 °C, 15 min), pH was adjusted to 5.6 ± 0.2 with NH_4_OH (Sigma-Aldrich, St. Luis, MO, USA). Then, to activate the calcium alginate cross-linking, a sterile and homogeneous suspension of CaHPO_4_ (20 mL; 0.5%, *w/v*) (Sigma-Aldrich, St. Luis, MO, USA) was added. The mixture was kept stirring for 30 min a room temperature. Then, the re-suspended probiotic biomass was added to form the control encapsulation solution. Finally, the bacterial suspensions with total viable counts between 10^8^ and 10^9^ CFU/mL were kept under constant stirring until spray-drying.

Viscosity. The viscosity of control encapsulation solutions was measured by a Digital Viscometer VISCO™—895 Package B (Atago Co., Ltd., Tokyo, Japan). The sample volume of 16 mL was kept at 21 °C.

### 2.4. Effect of CM or FM on Probiotic Survival After Spray-Drying and Viability During Storage

Effects of CM or FM were evaluated on probiotics (*B. infantis*, *B. longum*, *L. plantarum,* and *L. rhamnosus*) survival after spray-drying with an air inlet temperature at 130 °C and their viability during storage at 4, 25, and 37 °C for 90 days. CM or FM (0.4%, *w/v*) was mixed with sodium alginate (4.0%, *w/v*) and succinic acid (2.0%, *w/v*) in distilled water (100 mL) and sterilized (121 °C, 15 min). The pH was then adjusted to 5.6 ± 0.2 with NH_4_OH. A sterile, homogeneous suspension of CaHPO_4_ (20 mL; 0.5% *w/v*) was then added. The mixture was stirred for 30 min at room temperature. Then, the re-suspended probiotic biomass was added to form the encapsulation solution. Finally, the bacterial suspensions with total viable counts similar to item 2.3 were kept under constant stirring until spray-drying. The composition of the encapsulation solution is shown in Table 1.

Viscosity. The viscosity of encapsulation solutions supplemented with CM or FM was measured as previously described in item 2.3.

### 2.5. Spray-Drying

Drying assays were performed in a laboratory spray dryer unit (Büchi B290, Flawil, Switzerland). The process parameters were set as follows: inlet temperature = 130 °C; feed flow rate = 6 mL/min; air flow rate = 45 m^3^/h; outlet temperature = 72–75 °C. A one-fluid nozzle was used with a 0.7 mm orifice diameter. The probiotic microcapsules obtained by spray-drying were stored in hermetically sealed glass and stored at 4 °C until analysis. Each drying assay was carried out in triplicate. Survival after spray-drying and viability during storage of encapsulated bacteria was evaluated.

### 2.6. Analysis

#### 2.6.1. Physicochemical Characteristics of CM and FM

Chemical composition. The chemical composition of CM and FM was determined by AOAC methods [34]. The composition of monosaccharides and uronic acids was determined by high-performance liquid chromatography (HPLC), according to Sciarini et al. [35], with some modifications. Briefly, 100 mg of sample (CM or FM) was dissolved in 10 mL of sulfuric acid (1 M) and stirred at 95 °C for 24 h in a sealed tube to hydrolyze polysaccharide. The hydrolyzed suspension was cooled to ambient temperature and neutralized with NaOH (1 M). The volume of the suspension was adjusted to 50 mL with mili-Q water, the supernatant was recovered by centrifugation (10,000× *g* for 15 min), and the presence of particles was removed by filtration (0.22 μm). Then, samples were analyzed by HPLC using a Bio-Rad Animex HPX-42A column with an RI detector in an Alliance Waters e2695 Separation Module (Waters Inc., Milford, MA, USA). The sample (20 μL) was eluted with deionized water at 0.5 mL min^−1^.

Bulk density. The bulk density was measured according to Joshi et al. [36] with some modifications. Briefly, 2 g of CM or FM was transferred to a 10 mL graduated measuring cylinder with a lid. The cylinder was mounted on a shaker and agitated for 10 min. The weight and volume of the powder were recorded, and bulk density was expressed as kg/m^3^.

Color. The color of the CM or FM powder was measured using glass cuvettes and a spectrophotometer (NS800, 3NH Technology Co., Ltd., Guangzhou, China) set to operate with D65 lightning and a 10° observation angle. Color values were expressed as L* (black to white), a* (red to green), and b* (yellow to blue) parameters in the CIE system. The spectrophotometer was calibrated with black and white tiles before analysis, with the white calibration standard (L* = 96.62, a* = −0.09, b* = 1.09) as reference. The total color change (ΔE) was calculated from

ΔE* = [(L*_sample 1_ – L*_sample 2_)^2^ + (a*_sample 1_ – a*_sample 2_)^2^ + (b*_sample 1_ – b*_sample 2_)^2^] ^(1/2)^ (1)

FTIR-ATR spectroscopy. The IR spectra of CM and FM were obtained using Fourier Transform Infrared (FTIR) spectroscopy using the Jasco FTIR 4600 spectrophotometer (Jasco Corporation, Tokyo, Japan), equipped with an attenuated total reflection (ATR) accessory using a ZnSe crystal at a 45° incidence angle in a horizontal orientation. Spectra were collected in the range of 600 and 4500 cm^−1^, at a scan rate of 20 scans/s, with 4 cm^−1^ resolution at 25 °C.

#### 2.6.2. Enumeration of Viable Probiotics

Survival after drying and viability during storage of probiotics were determined by the standard plate count method. Briefly, 0.1 g of powder was diluted in 4.9 mL of sterile buffered peptone water (0.1%, *w/v*); the suspension was kept at 4 °C for 30 min to release the probiotics. The appropriate dilution of the probiotic suspension was seeded on MRS agar and incubated at 37 °C for 48 h. MRS agar supplemented with L-cysteine-HCl (0.05%, *w/v*) was applied for *B. infantis* and *B. longum* and incubated under anaerobic conditions.

Probiotic viability during storage was expressed as colony-forming units per gram (CFU/g) of dry powder, and the results were expressed as Log CFU/g. The survival percent after drying was calculated by Simpson et al. [37]:

(2) $$\mathrm{Survival}\;(\%)=(\;\frac{N}{N_{0}\;}\;)\times 100$$

where *N* is Log CFU/g of the spray-dried powder immediately after drying, and *N*_0_ is Log CFU/g of dry matter in the suspension fed to the dryer. The assay was carried out in triplicate.

#### 2.6.3. Storage of Spray-Dried Probiotics Powder

The dry powders were stored in sealed bottles at 4, 25, and 37 °C. Probiotic viability was determined every 7 days until 28 days were completed, and then every 15 days until 90 days.

The specific degradation rate (*k*, day^−1^) of probiotics microcapsules was calculated as a first-order reaction from

(3) $$k=\frac{Log\;\left(\frac{N_{0}}{N}\right)}{t}$$

where *N* is the probiotic count in a particular storage period (CFU/g), *N*_0_ is the probiotic count at the beginning of storage (CFU/g), and *t* is the storage time (days).

The activation energy (*Ea*) was determined from the Arrhenius equation

(4) $$Logk=Logk_{0}-\frac{Ea}{2.303\;R}\times \frac{1}{T}$$

where *k* specific rate of degradation (day^−1^), *Ea* (J · mol^−1^), *R* is the gas constant (8.321 J · mol^−1^ · K^−1^), and *T* is the absolute temperature (K) [38].

#### 2.6.4. Physical Properties of Probiotic Microcapsules

Moisture content. The residual moisture content of probiotic microcapsules was determined by oven drying at 105 °C until a constant weight was achieved and was repeated in duplicate.

Morphology. The morphology of probiotic microcapsules was observed by scanning electron microscope (SEM) SU3500 (JEOL Hitachi, Tokyo, Japan) at 20 KV, 30 Pa vacuum, and Backscattered Electron signal. The sample was dispersed over the sample holder equipped with a double-sided carbon type.

Particle size. The particle size of probiotic microcapsules was determined by laser diffraction (Particle size analyzer Shimadzu model SALAD-3101, Tokyo, Japan) at 25 °C using isopropyl alcohol as the continuous phase.

#### 2.6.5. Statistical Analysis

Analysis of variance (ANOVA) was applied to determine the significance of effects (*p* < 0.05). Differences between means were detected by the general linear model procedure, using Tukey’s test one-way analysis of variance (SPSS^®^ version 23).

## 3. Results

### 3.1. Physicochemical Characteristics of CM and FM

Chemical composition. The mucilage extraction yield was 7.90 ± 1.46 and 9.85 ± 0.86 for CM and FM, respectively (Table 2). The main component in CM and FM was the non-nitrogen extract (i.e., sugars, starch, and polymeric substance, excluding fiber), reaching 55.28% and 64.73%, respectively. CM samples have higher protein (13.14%), lipid (3.36%), and ash (12.82%) contents than FM samples. Monosaccharides of CM consisted mainly of glucose and rhamnose with ~2:1 glucose-to-rhamnose ratio. Moreover, glucuronic and galacturonic acids were not detected in CM samples, contrary to previous reports [39]. FM monosaccharides consisted mainly of glucose (37.50 ± 1.06%) and glucuronic acid (13.76 ± 1.08%), and lower concentrations of arabinose, galacturonic acid, mannose, and rhamnose. In this study, xylose, considered a neutral sugar, was not detected.

Viscosity. The viscosity of the control encapsulation solution was affected by the presence of CM or FM (Table 2). The viscosity of the CM-supplemented encapsulation solution was significantly (*p* < 0.05) higher (62%) than the control encapsulation solution (3.75 ± 0.14 mPa · s) and 39% higher than the FM-supplemented solution. Barbary et al. [40] and Timilsena et al. [39], evaluating aqueous solution (0.5%, *w/v*) of FM and CM, reported viscosities of 101.5 mPa · s and 1835.2 mPa · s, respectively. Therefore, the viscosities of the CM- and FM-supplemented encapsulation solution in this study decreased by 99.5% and 94.0%, respectively, compared to the corresponding 0.5% aqueous solutions of CM or FM. Bustamante et al. [33] reported that a 0.2% (*w/v*) FM encapsulation solution with 15% (*w/v*) maltodextrin had a viscosity of 7.87 ± 0.03 mPa · s, 22.6% higher than the FM-supplemented encapsulation solution used in the present study.

Bulk density. The bulk density of FM (686.28 ± 14.11 kg m^−3^) powder was higher (~37%) than that of CM powder (429.89 ± 7.96 kg m^−3^) (Table 2); the difference can be related to the higher CM moisture content.

Color. To the human eye, mucilage produced and released by chia seeds was beige, while the FM color was light brown. The color measurement (Table 2) showed that CM and FM are neutral powders with a slight tendency at lightness for FM (L* = 51.08). Both mucilage powders show a tendency to red and yellow hues for the values obtained from a* and b*, respectively.

**Figure 1:**
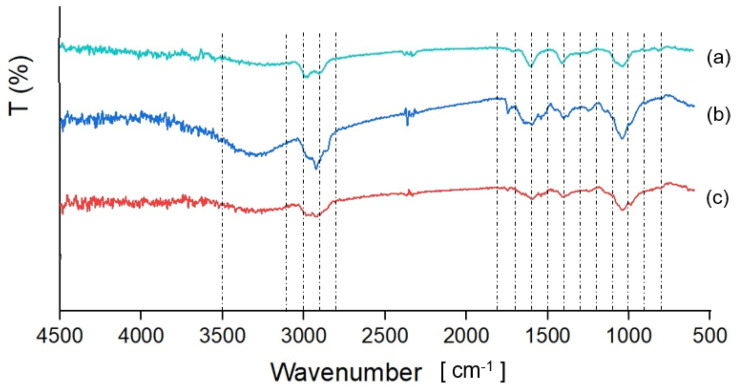
FTIR spectra of (**a**) commercial sodium alginate, (**b**) CM, and (**c**) FM.

FTIR-ATR spectroscopy. The FTIR-ATR was used to characterize the presence of specific functional groups in CM and FM—both extracted from seeds purchased in the local market—and to compare them with the spectral profile of sodium alginate. The overall FTIR-ATR spectra of CM and FM (Figure 1) show similar characteristics to those found in polymeric materials such as gums and mucilages, in the range from 3500 cm^−1^ to 900 cm^−1^ [41]. CM exhibits a broad band between 3500 and 3100 cm^−1^, corresponding to hydroxyl (-OH) stretching, which is characteristic of carbohydrate structure. This band is slightly detected in FM samples. Both CM and FM show peaks between 3100 and 2800 cm^−1^, attributed to the symmetric and asymmetric stretching and bending vibrations of methyl -C-H bond [42]. The peak observed between 1800 and 1700 cm^−1^ results from the stretching vibration of the carbonyl group (C=O), while the peak at 1600 cm^−1^ peak is due to NH group vibrations from secondary protein structures [43,44]. The peak at 1400 cm^−1^ is associated with the symmetrical stretching of carboxyl groups (-COO^-^) of uronic acids [39]. The presence of uronic acids is characteristic of seed mucilage and provides an anionic character to the molecule [45]. The peak between 1100 and 1000 cm^−1^ is associated with C–O–C stretching of 1, 4 glycosidic bonds and the elongation of C-O groups [41]. Finally, the peaks between 1400 cm^−1^ and 900 cm^−1^ indicate the presence of uronic acids and xylan-rich pectin polysaccharides [46].

On the other hand, the alginate sample presented a broad band between 3500 and 3100 cm^−1^ corresponding to hydroxyl (-OH) stretching [42,47], and the peak between 3100 and 2800 cm^−1^ represented CH_2_ stretching [48]. The peaks observed at 1600 and 1400 cm^−1^ correspond to asymmetric and symmetric stretching of the carboxyl group (-COO^−^) of uronic acid, respectively [49,50]. The peak between 1100 and 1000 cm^−1^ is attributed to the –C–O–C glycosidic linkage [49]. The peaks between 900 and 800 cm^−1^ represent stretching vibrations of C–H bonds [47], with the peak at 820 cm^−1^ being associated with the valence vibrations related to D-mannuronic acid [50].

### 3.2. Effect of CM or FM on Probiotic Survival After Spray-Drying and Viability During Storage

Recently, a new process has been developed in which particle formation, alginate cross-linking, and drying are carried out in a single step. We evaluated this new method and its effect on the survival of four probiotic strains after spray-drying and viability during storage. Then, the control encapsulation solution was supplemented with CM or FM prior to evaluating the probiotics’ survival and viability after spray-drying and during storage.

Results of probiotics survival (Table 3) showed high survival in the control encapsulation solution (>96%) as well as in those supplemented with CM or FM (86.7–98.7%). Supplementation with CM or FM significantly (*p* < *0.05*) enhanced the survival of *L. plantarum*, *B. infantis*, and *B. longum* compared to the survival of *L. rhamnosus* (Table 3). In contrast, the presence of FM significantly (*p* < *0.05*) reduced *L. rhamnosus* survival. Meanwhile, the control encapsulation solution significantly (*p* < *0.05*) enhanced the survival of *L. plantarum* compared to *B. longum*.

Each probiotic presented different survival rates when encapsulated by spray-drying in three different encapsulation solutions. The results show that *L. plantarum* had a significantly (*p* < 0.05) higher survival rate in the control and CM-supplemented encapsulation solutions (98%). Meanwhile, *B. longum* survival was significantly enhanced (*p* < 0.05) when the encapsulation solution was CM or FM-supplemented.

The viability of the four probiotic strains was evaluated during storage at 4 and 25 °C for 90 days after their encapsulation in a cross-linked alginate matrix with CM or FM. Furthermore, to simulate more demanding temperature conditions, which can occur in warm regions or during storage or transportation under uncontrolled conditions, the viability of the four probiotic strains was evaluated during storage at 37 °C for 90 days after the encapsulation process. At the beginning of storage, the viable probiotic count ranged between 11.77 ± 0.11 and 9.27 ± 0.04 Log CFU/g dry powder (Table 4).

**Figure 2:**
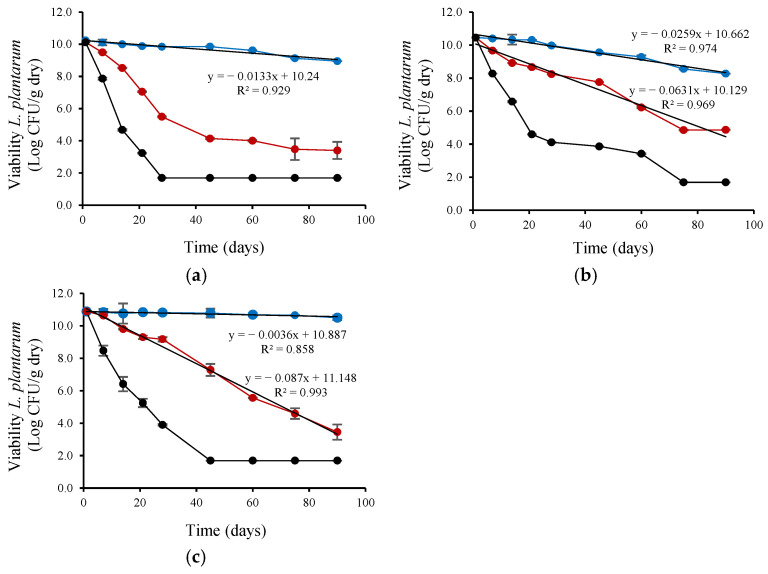
Viability during storage of *L. plantarum* encapsulated by spray-drying in control encapsulation solution added with (**a**) FM (0.4%, *w/v*), (**b**) CM (0.4%, *w/v*), (**c**) without CM or FM; for 90 days at (●) 4 °C, (●) 25 °C and (●) 37 °C.

**Figure 3:**
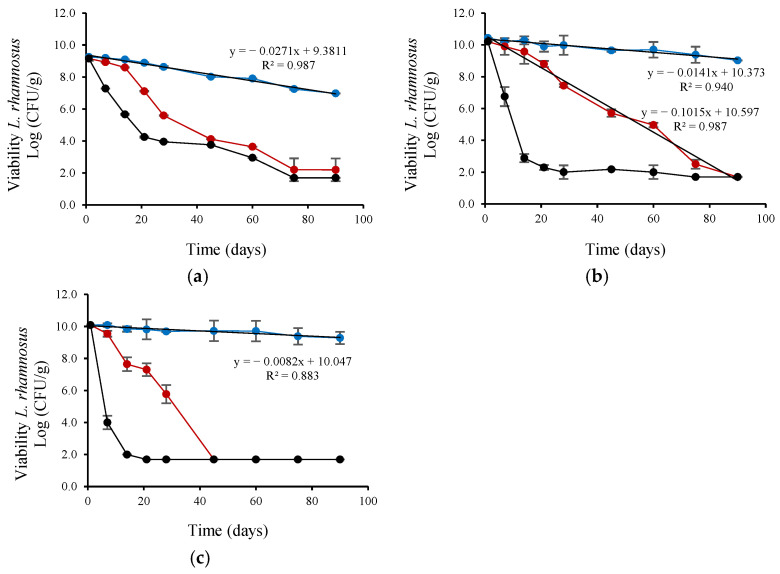
Viability during storage of *L. rhamnosus* encapsulated by spray-drying in control encapsulation solution added with (**a**) FM (0.4%, *w/v*), (**b**) CM (0.4%, *w/v*), (**c**) without CM or FM, for 90 days at (●) 4 °C, (●) 25 °C and (●) 37 °C.

**Figure 4:**
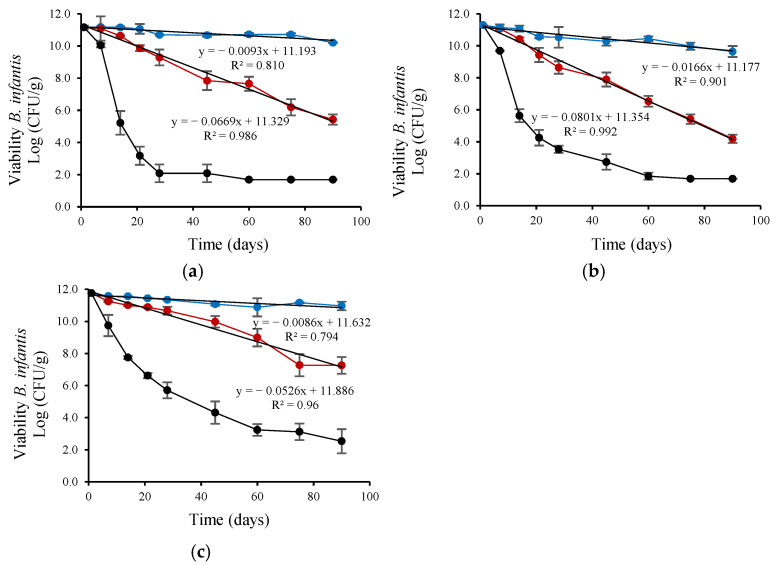
Viability during storage of *B. infantis* encapsulated by spray-drying in control encapsulation solution added with (**a**) FM (0.4%, *w/v*), (**b**) CM (0.4%, *w/v*), (**c**) without CM or FM; for 90 days at (●) 4 °C, (●) 25 °C and (●) 37 °C.

**Figure 5:**
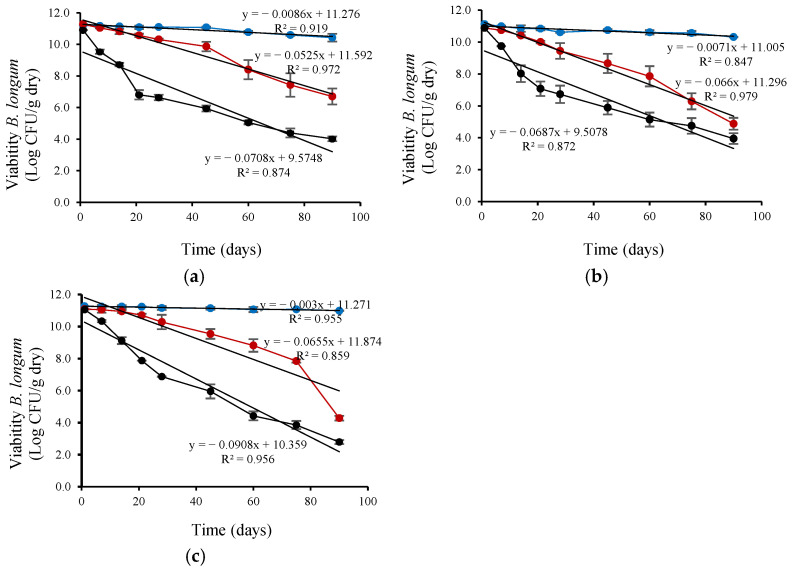
Viability during storage of *B. longum* encapsulated by spray-drying in control encapsulation solution added with (**a**) FM (0.4%, *w/v*), (**b**) CM (0.4%, *w/v*), (**c**) without CM or FM; for 90 days at (●) 4 °C, (●) 25 °C and (●) 37 °C.

*L. plantarum* viability (Figure 2) decreased linearly during refrigerated storage (4 °C). The viability reduction rate (based on the linear slope) was highest for *L. plantarum* encapsulated with CM, intermediate for FM, and lowest for the control (without CM or FM), with slopes of 0.0259, 0.0133, and 0.0036, respectively. *L. plantarum* encapsulated with CM also showed a linear decrease in viability during ambient storage (25 °C) but at a lower rate (0.0631) than control (0.087). *L. rhamnosus* viability (Figure 3) also decreased linearly during refrigerated storage (4 °C). The viability reduction rate (based on the linear slope) was highest for *L. rhamnosus* encapsulated with FM, intermediate for CM, and lowest for the control (without CM or FM), with slopes of 0.0271, 0.0141, and 0.0082 slope, respectively. *L. rhamnosus* encapsulated with CM also showed a linear decrease in viability during ambient storage (25 °C) but at a higher rate (0.1015; ~7-fold) than during storage at 4 °C. As shown in Figure 4, *B. infantis* viability decreased linearly during refrigerated storage (4 °C). The viability reduction rate (based on the linear slope) was highest for *B. infantis* encapsulated with CM, intermediate for FM, and lowest for control (without CM or FM), with slopes of 0.0166, 0.0093, and 0.0086, respectively. A similar trend was observed during ambient storage (25 °C), but at a higher rate—4.8-, 7.2- and 6.1-fold increases for CM, FM, and control, respectively. The corresponding slopes were 0.0801, 0.0669, and 0.0526 for CM, FM and control, respectively. In Figure 5, *B. longum* viability also decreased linearly during refrigerated storage (4 °C). The viability reduction rate (based on the linear slope) was highest for *B. longum* encapsulated with FM, intermediate for CM, and lowest for control (without CM or FM) with slopes of 0.0086, 0.0071, and 0.003 slope, respectively. The rate of *B. longum* viability reduction was also linear during ambient storage (25 °C) but followed a different order, with the highest reduction rate for CM, followed by control and FM, with slopes of 0.066, 0.655, and 0.0525, respectively. Interestingly, *B. longum* viability in the control also decreased linearly during storage at 37 °C, with a slope of 0.0908, a ~30-fold reduction rate compared to storage at 4 °C.

As shown in Figure 2, Figure 3, Figure 4 and Figure 5 and Table 4, storage temperature was an important factor affecting the viability of spray-dried probiotics. During storage at 4 °C, viability decreased slightly, and after 90 days, the viable probiotic count in the microcapsules ranged between 6.99 and 10.99 Log CFU/g dry powder. This represented a viability loss of 2.28% and 0.08% for *L. rhamnosus* encapsulated with FM and *B. longum* encapsulated in the control solution, respectively. In addition, *B. longum* viability was significantly (*p* < 0.05) higher when encapsulated in the control solution. Table 4 also shows that the recorded viability values were higher than the recommended threshold (6 Log CFU/g of product) for probiotics to confer health benefits for consumers [51]. The viable probiotic count during storage at 25 °C showed a greater reduction compared to storage at 4 °C. At 25 °C, *L. rhamnosus* was the most affected strain, with viable cell counts below 6 log CFU/g of dry powder after 45 days of storage and dropping to less than 2.2 log CFU/g after 90 days (Figure 3). The viability of *B. infantis* encapsulated in the control solution and *B. longum* encapsulated in the presence of FM was significantly (*p* < 0.05) higher after 90 days of storage at 25 °C, reaching values greater than 6 Log CFU/g of product. The number of viable bacteria decreased considerably at the high storage temperature (37 °C), showing a greater reduction than at 25 °C for all assays (Figure 2, Figure 3, Figure 4 and Figure 5). After 21 days of storage, *L. plantarum*, *L. rhamnosus*, and *B. infantis* were the most affected strains, presenting viable counts below 6 Log CFU/g dry powder (Figure 2, Figure 3 and Figure 4). In contrast, *B. longum* counts remained above 6.81 Log CFU/g dry powder for all encapsulation solutions (Figure 5). After three months of storage, viable cell counts of *B. longum* were significantly (*p* < 0.05) higher, reaching 4.02 and 4.00 Log CFU/g dry powder when encapsulated in the presence of FM and CM, respectively. However, these values are still below the recommended levels for providing health benefits to consumers.

### 3.3. Survival Rates of Probiotic Strains During Storage

**Figure 6:**
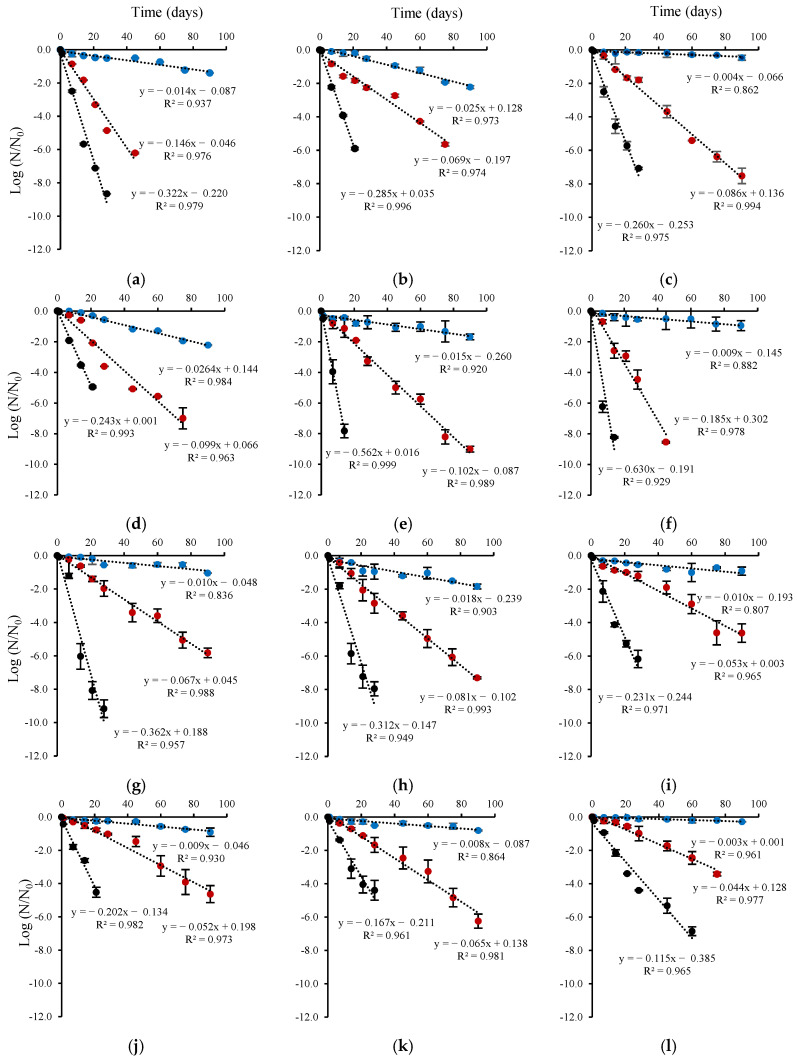
Survival rates of microencapsulated probiotic strains as a function of the encapsulation solution and storage temperature. (**a**–**c**) *L. plantarum*, (**d**–**f**) *L. rhamnosus*, (**g**–**i**) *B. infantis* and (**j**–**l**) *B. longum*. Encapsulation solution supplemented with (**a**,**d**,**g**,**j**) FM, (**b**,**e**,**h**,**k**) CM, and (**c**,**f**,**i**,**l**) control. Storage temperature: (●) 4 °C, (●) 25 °C and (●) 37 °C.

The viability of the probiotic microcapsules was monitored during 90 days of storage. In addition, the dependence of probiotic strain survival rates on storage temperature (4, 25, and 37 °C) was determined (Figure 6). The rate constants for probiotic inactivation during storage were calculated according to a first-order kinetics model. The first-order model showed a good fit to the data (*R*^2^ = 0.807–0.999) to the data, particularly at storage temperatures of 25 and 37 °C.

The control encapsulation solution increased the storage stability of dehydrated probiotic strains (*L. plantarum*, *L. rhamnosus*, *B. infantis*, and *B. longum*) compared to the encapsulation solution supplemented with CM or FM at 4 °C. The control encapsulation solution was effective at the lower temperature (4 °C), showing lower *k* values, between 0.003 day^−1^ for *B. longum* to 0.010 day^−1^ for *B. infantis* (Figure 6). Moreover, the FM-supplemented encapsulation solution also provides stability to *B. infantis* during storage at 4 °C, with a *k* value of 0.01 day^−1^. *B. longum*, encapsulated in the control solution as well as in those supplemented with CM and FM, was the least susceptible to viability loss during storage at 4°C, with *k* values ranging from 0.003 day^−1^ to 0.010 day^−1^ (Figure 6). The CM-based *L. plantarum* and FM-based *L. rhamnosus* microcapsules were the most susceptible to storage-related viability loss, showing 3-fold and 10-fold reductions in survival rates at 25 °C and 37 °C, respectively, compared to 4 °C. At 37 °C, probiotic viability loss was significant, and the composition of the encapsulation solution had a major effect on viability. The activation energies (*E*a) of the probiotics (Table 5) were calculated by fitting the inactivation rate constant data to the Arrhenius equation. *L. plantarum*, *L. rhamnosus*, and *B. longum* powders based on the control encapsulation solution were more susceptible to thermal damage, with *E*a values of 91.80, 92.98, and 80.21 kJ/mol K, respectively. In contrast, the lower *E*a values observed for CM-based *L. plantarum* and *B. infantis* (50.00 and 60.00 kJ/mol K) and FM-based *L. rhamnosus* and *B. longum* (47.41 and 66.00 kJ/mol K), indicate greater thermal stability, which can result in improved viability under temperature variations.

### 3.4. Physical Properties of Dry Probiotic Microcapsules

Moisture content: Spray-dried powders showed high residual moisture contents (12.7–16.9%) (Table 6), which were higher than those previously reported. Tan et al. [52] reported ~8% moisture content for encapsulation solutions formulated with sodium alginate only (2%, *w/v*) and for formulations with CaCl_2_ (10 mM) and sodium alginate (2%, *w/v*), both dried at an inlet air temperature of 120 °C. Strobel et al. [31] reported ~7% moisture content for an encapsulation solution with CaHPO_4_ (0.1 or 0.5%, w/w) and sodium alginate (2%, w/w), using an inlet air temperature of 130 °C.

**Figure 7:**
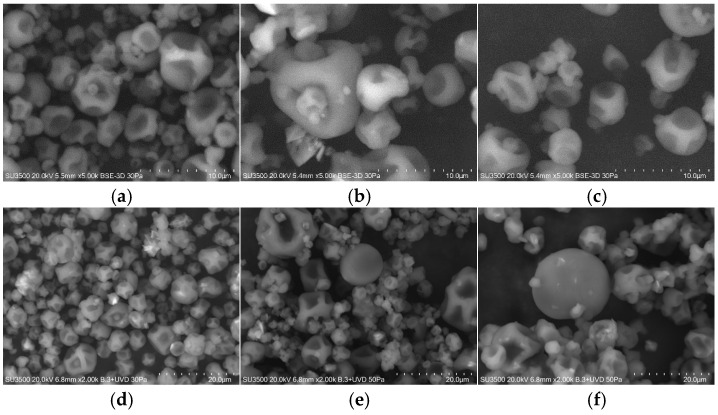
SEM micrographs of dry microcapsules containing *L. rhamnosus* ATCC53103 storage at 4 °C after 1 day (**a**–**c**), and (**d**–**f**) 90 days. Encapsulated cell with sodium alginate, succinic acid, and CaHPO_4_ (**a**,**d**) CM (0.4%, *w/v*), (**b**,**e**) FM (0.4%, *w/v*), and (**c**,**f**) control (0.0% seeds mucilage) dried with an inlet air temperature of 130 °C.

Morphology**:** Figure 7 shows the scanning electron micrographs of microcapsules containing *L. rhamnosus* ATCC 53103, encapsulated in the presence of CM (Figure 7a,d), FM (Figure 7b,e) or in the control encapsulation solution (without CM or FM) (Figure 7c,f), after 1-and 90-days storage at 4 °C. The microcapsules were spherical, with variable diameters, concavities, and surface deflations without evidence of cracks or fissures.

Particle size: The particle size analysis revealed a monomodal distribution for all assays, i.e., for microcapsules containing *L. plantarum*, *L. rhamnosus*, *B. infantis*, and *B. longum* encapsulated in the control solution, as well as in the solutions supplemented with CM or FM, as shown in Figure 7 for *L. rhamnosus* microcapsules. Furthermore, the microcapsules were fine powders, with mean particle diameters ranging from 7.1 to 11.8 for the encapsulation solutions supplemented with CM and FM (Table 6), respectively, values within the typical size range of powders produced from spray-drying (10–50 μm) [53]. The presence of FM in the encapsulation solution significantly (*p* < 0.05) affected the particle size by increasing the diameter compared to CM microcapsules.

## 4. Discussion

In this study, we evaluated the effect of a cross-linked alginate matrix coating material supplemented with CM or FM on probiotics viability after spray-drying and during storage. As a first step, the extraction yields of CM and FM were determined, and the results were consistent with previous reports. However, the extraction yield depended on the seed type and experimental factors, such as temperature, rotation speed, pH of the extraction liquid, water-to-seed ratio, ionic strength, extraction method, and time [54,55]. High temperatures are preferred, since they promote the polysaccharides solubilization in water and therefore increase the extraction yield [56].

Seed mucilage is a complex polysaccharide containing several sugars in its structure; the heteropolysaccharide CM consists of D-xylose, D-mannose, L-arabinose, D-glucose, galacturonic acid, and glucuronic acid [39,57].

FM is a heterogeneous polysaccharide composed of neutral arabinoxylan, consisting of D-arabinose, D-xylose, and D-galactose, and acidic rhamnogalacturonan, composed of L-rhamnose, L-fucose, L-galactose, and D-galacturonic acid [26]. Therefore, the differences detected in other studies in relation to the chemical composition could be due to the variety and origin of the seed and the variation in the growth conditions [39].

Although viscosity depends on mucilage extraction conditions, seed type, and the experimental parameters used for its determination, the difference between both encapsulation solutions was significant, reaching 94.5%. Timilsena et al. [39] and Kaewmanee et al. [58] suggest that the viscosity of a mucilage solution depends on the composition and chemical structure of its polysaccharides. In this study, this difference can also be attributed to the higher solids content in the encapsulation solution supplemented with CM, as CM contains higher concentrations of proteins, lipids, crude fiber, and ash than FM (Table 2). According to Maskan and Gogus [59], this higher viscosity can considerably restrict the intermolecular motion caused by hydrodynamic forces and interfacial film formation. Furthermore, the results also show significant differences between the viscosity of the mucilage solution (0.5%, *w/v*) and the encapsulation solutions supplemented with FM or CM of this study. This difference may be related to the presence of calcium salts, which promote cross-linking of alginate in the encapsulation solution. The saline solution can lead to the formation of high molecular weight aggregates, with compact and homogeneous structures in the mucilage, due to the contraction of the polysaccharide molecules, thereby lowering viscosity. This effect depends on the type of salt, where viscosity reduction is more pronounced in the presence of divalent ions (CaCl_2_) compared to monovalent ions (NaCl) [60,61].

Color depends on various factors such as the seed color, powder structure, packing density, severity of the extraction conditions, and the drying process of the mucilage [62]. In our study, the color of the extracted mucilage was influenced by both the seed and extraction conditions, where the latter required the use of distilled water between 80 and 95 °C. Moreover, extraction processes involving high temperatures and prolonged exposure times can result in brown mucilage due to enzymatic browning, which affects polysaccharide properties [63]. According to the color measurements, the total color difference between the two mucilage (CF and FM) powders was 2.93, indicating noticeable color differences [64] (ΔE* values > 2).

Spray-drying is the most studied and widely used industrial technique for encapsulating probiotics. However, the high processing temperatures and low moisture content of the final product can cause cell damage and loss of activity, thereby affecting the viability of these microorganisms. This has led to the study of new processes or modifications of those currently used to achieve high survival rates and maintain the metabolic activity of microorganisms. Each encapsulation process must demonstrate microorganisms’ survival after the process, during storage, and throughout transit in the gastrointestinal tract. In addition, it must ensure the survival of viable cells (at least 6 Log CFU/g or mL of product) at the time of consumption so that probiotics can exert their beneficial effects on health. Our results show high survival values for the four probiotics in the three-encapsulation solutions, suggesting that *L. plantarum*, *L. rhamnosus*, *B. infantis*, and *B. longum* were thermally protected from cell inactivation during the spray-drying process. The level of protection achieved for each probiotic is related to its intrinsic characteristics and to the properties of the encapsulation solution, such as solids concentration and viscosity, among other factors. Overall, probiotics survival was higher when the encapsulation solution was supplemented with CM, although this difference was not significant in all cases. This condition may be related to CM’s chemical composition (high concentration of proteins, lipids and crude fiber) and its high viscosity, providing high cellular protection against thermal damage.

The storage temperature was an important parameter affecting the viability of spray-dried probiotics. Viable probiotics concentration was maintained under refrigeration (7–11 Log CFU/g of product) until the end of storage (3 months), demonstrating good storage stability of the probiotic microcapsules, associated with the greater stability of the encapsulation solutions at low temperatures. These concentrations correspond with FAO/WHO [2] recommended levels to achieve a positive effect on consumer health. In addition, significant (*p* < 0.05) viability loss occurred at different rates at high temperatures (25 °C and 37 °C) for all conditions, except for *B. longum* microcapsules, which, after 45 days, presented levels ~6 Log CFU/g of product. Storage temperature can affect probiotics viability through two mechanisms: increased metabolic activity rate due to increased temperature and modification of water molecular mobility. It can also be considered that the greater metabolic activity of the cell involves the production of metabolites, which can cause cellular inactivation.

*L. plantarum* and *B. infantis* encapsulated with FM presented superior stability during refrigerated storage (4 °C) compared to CM encapsulation. *B. longum* encapsulated with FM also showed greater storability than CM encapsulation and control during ambient (25 °C) storage based on viability and rate reduction (Figure 2, Figure 3, Figure 4 and Figure 5). In contrast, CM encapsulation was more suitable for *L. rhamnosus* and *B. longum* during refrigerated storage (4 °C) instead of FM based on their viability (Figure 2, Figure 3, Figure 4 and Figure 5).

The protection level of the encapsulation solutions can be expressed in terms of specific survival rate, where the control encapsulation solution was more effective or stable during low temperature (4 °C) storage with a lower *k* value, which is related to higher viable cell concentration after 90 days (Table 4). Probiotic viability loss was significant at a higher temperature (37 °C), and a high *k* value indicates that the encapsulation solutions were less stable, correlating with low viability after 14 days of storage for *L. plantarum*, *L. rhamnosus*, and *B. infantis*. *L. rhamnosus* was most susceptible to high storage temperatures, which may be related to its intrinsic properties, showing less resistance to high temperatures and low humidity environments, characteristics that the encapsulation solution could not supply. The *E*a analysis shows that powders based on the control encapsulation solution were highly susceptible to thermal damage and, therefore, more unstable than powders supplemented with CM or FM. The stability of mucilage-based powders is due to the thermal stability of CM and FM, which exhibit thermal degradation between 280 and 360 °C and 200–350 °C, respectively, as determined by Timilsena [39] and Dubois [65].

The differences observed in the moisture content of probiotic microcapsules, compared to other studies, can be attributed to the methodology used in this study. Tan et al. [52] and Strobel et al. [31] determined moisture content at 65 °C after 3 days of drying, and such prolonged drying times can influence the final moisture levels of the sample. In addition, calcium alginate particles can absorb and retain moisture. Spray-dried powder products are generally considered to be of good quality when the residual moisture content is less than 4% [66]. This moisture level helps ensure product stability and longer shelf life during storage by limiting chemical reactions due to reduced water availability. It also facilitates proper handling, packaging, and transportation. The probiotic microcapsules in this study remained viable after 90 days of storage at 4 °C (Figure 7), even with a moisture content (12.7–16.9%, Table 6) higher than the recommended level. This represents an advantage over products such as yogurt, which contain high concentrations of non-microencapsulated free lactic acid bacteria but have a relatively short shelf life of 2 to 4 weeks. Microencapsulation protects the probiotics during storage, enhances product stability, and extends shelf life.

**Figure 8:**
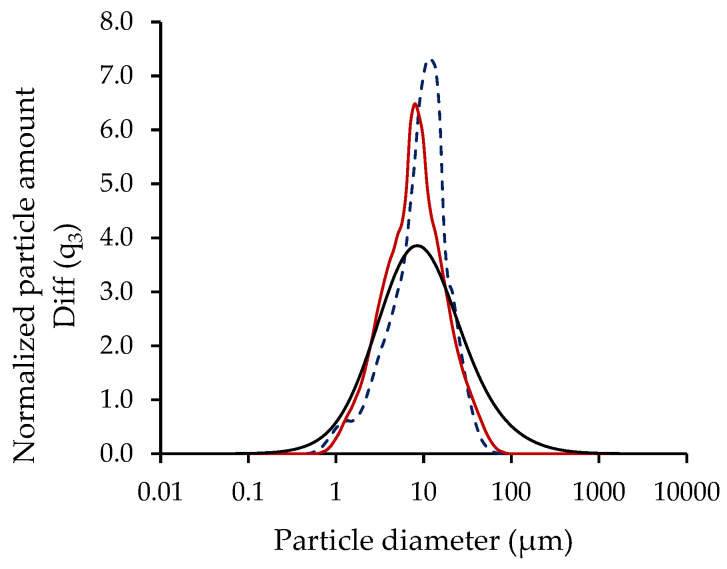
Particle size distribution of dry microcapsules produced with *L. rhamnosus* encapsulated in control solution (**―**), solution supplemented with CM (**―**) or FM (**- - -**).

Overall, probiotic microcapsules showed varying degrees of the “flat ball” effect or surface cavities as a result of heat penetration [67] and rapid water evaporation [68]. The larger size of the microcapsules supplemented with FM can be related to their higher moisture content (Table 6). In addition, agglomeration can be possible due to the presence of calcium salts, which promote cross-linking of the alginate, as demonstrated by the shoulders in the particle size distribution curves of the microcapsules supplemented with FM and CM (~1 μm and 50 μm), respectively (Figure 7) [31]. Agglomeration can also result from small microcapsules being trapped within larger cavities, artificially increasing the measured particle diameter (Figure 8). The fine powders obtained in this study can help reduce the impact on sensory texture when incorporated into a model food matrix.

Although no significant differences were observed in the survival and viability of probiotics encapsulated in a cross-linked alginate matrix supplemented with CM or FM compared to the control encapsulation solution, the use of these compounds is justified for several reasons. Both CM and FM are natural, hydrophilic, easily extracted, and biodegradable polysaccharides. Moreover, they contain components with prebiotic properties, enabling their metabolism by probiotics and inhibiting the growth of pathogenic microorganisms, thereby promoting intestinal health. Several studies have demonstrated that their consumption can have beneficial effects on human health. In the food industry, these polysaccharides are widely used as functional additives due to their gelling, thickening, and emulsifying properties, which help improve the texture, flavor, and shelf life of products. Furthermore, today’s consumers are increasingly informed and tend to prefer products that not only provide an adequate nutritional profile but also offer additional health benefits.

Finally, the findings of this study raise new research questions. In particular, it is important to evaluate the effect of supplementing the cross-linked alginate matrix with higher concentrations of CM or FM on probiotics viability, as well as to evaluate the degree of protection that this matrix could offer under simulated gastrointestinal conditions.

## 5. Conclusions

*L. plantarum*, *L. rhamnosus*, *B. infantis*, and *B. longum* can be microencapsulated by spray-drying in a cross-linked alginate matrix with or without CM and FM supplementation. All probiotics showed high survival rates after spray-drying, reaching values > 85% for the control encapsulation and those supplemented with CM or FM. The probiotic viability was affected by storage temperature and the refrigeration condition, allowing high levels (>6 Log CFU/g) of viability for over 90 days. In contrast, at 37 °C, the recommended viability for probiotics to exert health benefits does not exceed 14 days. The addition of CM and FM enabled higher thermal stability of the control encapsulation solution (composed of sodium alginate, succinic acid, CaHPO_4_, and probiotics). Therefore, the microencapsulation of probiotics in cross-linked alginate matrices supplemented with chia or flaxseed mucilage via spray-drying emerges as a viable alternative for incorporating polysaccharides with prebiotic components. These compounds can stimulate not only the growth of beneficial bacteria and the inhibition of pathogenic microorganisms but also exhibit functional properties that can enhance the stability and efficacy of the probiotic product.

## Figures and Tables

**Table 1:** Composition of formulations used for the encapsulation of probiotics by spray-drying in cross-linked alginate matrices supplemented with chia seed mucilage (CM) and flaxseed mucilage (FM).

Composition	Encapsulation Solution		
	Control	CM	FM
Sodium alginate (4% *w/v*)	x	x	x
Succinic acid (2%, *w/v*)	x	x	x
NH_4_OH	x	x	x
CaHPO_4_ (0.5%, *w/v*)	x	x	x
Chia seed mucilage (0.4%, *w/v*)		x	
Flaxseed mucilage (0.4%, *w/v*)			x

**Table 2:** Chemical composition of chia seed (CM) and flaxseed mucilage (FM).

Constituents	Unit	CM	FM
Yield ^a^	%	7.90 ± 1.46	9.85 ± 0.86
Moisture	%	11.29	9.23
Protein	%	13.14	12.12
Fat	%	3.36	0.62
Crude fiber	%	4.11	n.d.
Ash	%	12.82	11.85
Non-nitrogenous extract	%	55.28	66.18
Monosaccharides ^b^			
Arabinose	%	n.d.	1.54 ± 0.08
Fucose	%	n.d.	n.d.
Galacturonic acid	%	n.d.	2.28 ± 0.28
Glucose	%	36.80 ± 1.83	37.50 ± 1.06
Glucuronic acid	%	n.d.	13.76 ± 1.08
Mannose	%	n.d.	2.79 ± 0.15
Rhamnose	%	17.94 ± 0.85	4.62 ± 0.41
Xylose	%	n.d.	n.d.
Energy	Kcal/100 g	303.92	318.78
Bulk density ^c^	kg/m^3^	429.89 ± 7.96	686.28 ± 14.11
Color ^b^			
L*		48.15 ± 0.53	51.08 ± 0.62
A*		4.48 ± 0.22	4.65 ± 0.33
B*		17.76 ± 0.57	17.43 ± 0.50
Viscosity encapsulating solution added with 0.4% CM or FM ^c^	mPa·s	9.91 ± 0.30	6.09 ± 0.32

n.d. = not determined. ^a^: Data are average ± standard deviation of ten analysis. ^b^: Data are average ± standard deviation of three samples. ^c^: Data are average ± standard deviation of four samples.

**Table 3:** Effect of chia seed (CM) or flaxseed (FM) mucilage on probiotic survival after encapsulation by spray-drying at 130 °C.

Probiotic Strain	Survival ^††^ (%)		
	CM	FM	Control
*L. plantarum*	98.66 ± 0.19 a A	95.53 ± 0.50 b A	98.43 ± 0.47 a A
*L. rhamnosus*	92.06 ± 2.91 ab B	86.65 ± 3.19 b B	97.50 ± 0.69 a AB
*B. infantis*	96.61 ± 0.53 ab A	94.03 ± 2.87 b A	98.12 ± 0.26 a AB
*B. longum*	98.45 ± 0.37 a A	98.39 ± 0.65 a A	96.59 ± 0.80 b B

^††^: Mean values of three replicates. Means in a row followed by different lowercase letters are significantly different by Tukey’s test at the 5% level. Means in a column followed by different capital letters are significantly different by Tukey’s test at the 5% level.

**Table 4:** Effect of the composition of encapsulation solution on probiotics viability during storage at 4, 25, and 37 °C during 1 and 90 days after spray-drying.

Soluble Fiber ^†^	Viability (Log CFU/g Powder)			
	Day 1	Day 90		
	4 °C	4 °C	25 °C	37 °C
	*L. plantarum*			
CM	10.45 ± 0.08 d	08.27 ± 0.10 f	4.87 ± 0.30 bc	1.69 ± 0.00 c
FM	10.24 ± 0.01 d	08.95 ± 0.00 e	3.40 ± 0.34 e	1.69 ± 0.00 c
Control	10.89 ± 0.01 c	10.50 ± 0.22 abc	3.45 ± 0.30 de	1.69 ± 0.00 c
	*L. rhamnosus*			
CM	10.44 ± 0.05 d	09.02 ± 0.00 e	1.69 ± 0.01 f	1.69 ± 0.00 c
FM	09.27 ± 0.04 e	06.99 ± 0.03 g	2.19 ± 0.50 f	1.69 ± 0.00 c
Control	10.11 ± 0.07 d	09.29 ± 0.27 de	1.69 ± 0.00 f	1.69 ± 0.00 c
	*B. infantis*			
CM	11.33 ± 0.30 b	09.64 ± 0.41 d	4.25 ± 0.21 cde	1.69 ± 0.00 c
FM	11.19 ± 0.09 bc	10.21 ± 0.02 c	5.49 ± 0.30 b	1.69 ± 0.00 c
Control	11.77 ± 0.11 a	10.97 ± 0.19 ab	7.45 ± 0.32 a	2.53 ± 0.56 b
	*B. longum*			
CM	10.89 ± 0.01 bc	10.32 ± 0.02 c	4.91 ± 0.12 bc	4.00 ± 0.24 a
FM	10.94 ± 0.05 bc	10.47 ± 0.19 bc	6.76 ± 0.38 a	4.02 ± 0.13 a
Control	11.07 ± 0.04 bc	10.99 ± 0.01 a	4.32 ± 0.27 cd	2.79 ± 0.09 b

^†^ CM: Chia seed mucilage, FM: Flaxseed mucilage. Different letters in the same column indicate significant differences by Tukey’s one-way analysis of variance (*p* < 0.05).

**Table 5:** Effect of the wall composition on activation energy (*E*a) of the probiotic strain.

Probiotic Strain	Spray-Drying Medium ^†^	*E*a (kJ/mol K)	*R* ^2^
*L. plantarum*	C/CM	50.02	0.924
	C/FM	69.13	0.991
	C	91.80	0.994
*L. rhamnosus*	C/CM	76.31	0.977
	C/FM	47.41	0.994
	C	92.98	0.997
*B. infantis*	C/CM	60.05	0.977
	C/FM	75.60	0.977
	C	66.153	0.978
*B. longum*	C/CM	66.19	0.999
	C/FM	66.00	0.988
	C	80.21	0.993

^†^: CM = Chia seed mucilage, FM = Flaxseed mucilage, C = Control encapsulation solution. Composition control encapsulation solution: Sodium alginate, succinic acid, CaHPO_4_.

**Table 6:** Residual moisture content and particle size of probiotic microcapsules after encapsulation by spray-drying at 130 °C.

Probiotic Strain	Moisture ^†^ (%)			Particle Size ^††^ (μm)		
	CM	FM	Control	CM	FM	Control
*L. plantarum*	16.54 ± 0.48	16.91 ± 0.67	16.25 ± 0.27	8.50 ± 0.53 b	10.59 ± 0.66 a	8.22 ± 1.00 b
*L. rhamnosus*	15.03 ± 0.72	16.70 ± 0.85	15.33 ± 0.71	8.50 ± 0.53 b	11.82 ± 0.74 a	9.87 ± 1.08 ab
*B. infantis*	14.96 ± 0.68	14.03 ± 0.32	13.76 ± 0.61	7.07 ± 0.00 b	11.40 ± 0.74 a	9.53 ± 1.25 a
*B. longum*	13.25 ± 0.35	12.66 ± 0.31	14.08 ± 0.51	8.50 ± 0.53 b	10.21 ± 0.66 a	9.49 ± 0.59 ab

CM = Chia seed mucilage, FM = Flaxseed mucilage, CM: 0.4%, *w/v*, FM: 0.4%, *w/v*. ^†^: Mean values of four replicates. ^††^: Mean values of three replicates. Means in a row followed by different lowercase letters are significantly different by Tukey’s test at the 5% level.

## Data Availability

The original contributions presented in the study are included in the article, further inquiries can be directed to the corresponding author.
